# HER2 gene amplification and EGFR expression in a large cohort of surgically staged patients with nonendometrioid (type II) endometrial cancer

**DOI:** 10.1038/sj.bjc.6604814

**Published:** 2008-12-16

**Authors:** G E Konecny, L Santos, B Winterhoff, M Hatmal, G L Keeney, A Mariani, M Jones, C Neuper, B Thomas, L Muderspach, D Riehle, H-J Wang, S Dowdy, K C Podratz, M F Press

**Affiliations:** 1 Division of Gynecologic Surgery, Department of Obstetrics and Gynecology, Mayo Clinic, Rochester, MN, USA; 2 Division of Hematology–Oncology, Department of Medicine, David Geffen School of Medicine, University of California Los Angeles, Los Angeles, CA, USA; 3 Women's Cancer Program, Department of Obstetrics and Gynecology, Microbiology and Pathology, Norris Comprehensive Cancer Center, University of Southern California, Keck School of Medicine, Los Angeles, CA, USA; 4 Division of Anatomic Pathology, Department of Laboratory Medicine and Pathology, Mayo Clinic, Rochester, MN, USA; 5 Department of Biomathematics and Biostatistics, David Geffen School of Medicine, University of California Los Angeles, Los Angeles, CA, USA

**Keywords:** HER-2/neu, EGFR, endometrial cancer, lapatinib, trastuzumab

## Abstract

Type II endometrial cancers (uterine serous papillary and clear cell histologies) represent rare but highly aggressive variants of endometrial cancer (EC). HER2 and EGFR may be differentially expressed in type II EC. Here, we evaluate the clinical role of HER2 and EGFR in a large cohort of surgically staged patients with type II (nonendometrioid) EC and compare the findings with those seen in a representative cohort of type I (endometrioid) EC. In this study HER2 gene amplification was studied by fluorescence *in situ* hybridisation (FISH) and EGFR expression by immunohistochemistry. Tissue microarrays were constructed from 279 patients with EC (145 patients with type I and 134 patients with type II EC). All patients were completely surgically staged and long-term clinical follow up was available for 258 patients. The rate of HER2 gene amplification was significantly higher in type II EC compared with type I EC (17 *vs* 1%, *P*<0.001). HER2 gene amplification was detected in 17 and 16% of the cases with uterine serous papillary and clear cell type histology, respectively. In contrast, EGFR expression was significantly lower in type II compared with type I EC (34 *vs* 46%, *P*=0.041). EGFR expression but not HER2 gene amplification was significantly associated with poor overall survival in patients with type II EC, (EGFR, median survival 20 *vs* 33 months, *P*=0.028; HER2, median survival 18 *vs* 29 months, *P*=0.113) and EGFR expression retained prognostic independence when adjusting for histology, stage, grade, and age (EGFR, *P*=0.0197; HER2, *P*=0.7855). We conclude that assessment of HER2 gene amplification and/or EGFR expression may help to select type II EC patients who could benefit from therapeutic strategies targeting both HER2 and EGFR.

Endometrial carcinoma (EC) is the most common malignancy of the female reproductive tract. Based on pathological and clinical features, endometrial cancers are classified into two types. Endometrioid or Type I EC, represents the majority of EC cases, is oestrogen-related, usually arises in the setting of endometrial hyperplasia, and tends to be biologically less aggressive (Hecht and Mutter, 2006). Nonendometrioid or Type II EC, predominantly uterine serous papillary carcinoma and clear cell endometrial carcinoma, accounts for approximately 10% of ECs, is not oestrogen-related, arises from atrophic endometrium, and frequently presents in advanced stages with 5-year survival rates, on average, between 30 and 40% (Abeler and Kjorstad, 1991; Goff *et al*, 1994; Slomovitz *et al*, 2003). Extra-uterine disease is often found in these patients even in the absence of myometrial invasion (Abeler 1991; Goff *et al*, 1994; Bristow *et al*, 2001). Therefore, comprehensive surgical staging is recommended for all patients with type II EC regardless of the depth of myometrial invasion of the tumour (Chan *et al*, 2003). These different clinical features are paralleled by genetic distinctions. Type II ECs carry mutations of independent sets of genes compared with type I EC. Type I EC is associated with mutations in the *PTEN* tumour suppressor gene and defects in DNA mismatch repair (Lax *et al*, 2000, Hecht and Mutter, 2006). In contrast, p53 mutations, which are not usually seen in type I EC have been identified in most cases of type II EC (Acharya *et al*, 2005). Moreover, HER2 amplification/overexpression has been associated with type II EC (Hetzel *et al*, 1992; Santin *et al*, 2005; Morrison *et al*, 2006; Grushko *et al*, 2008). However, the exact frequency of HER2 amplification/overexpression in type II EC remains controversial. HER2 gene amplification has been reported to occur in 6 out of 28 (21%), 17 out of 58 (29%), or 11 out of 26 (42%) of patients with uterine serous papillary cancer (Santin *et al*, 2005; Morrison *et al*, 2006; Grushko *et al*, 2008), and HER2 protein overexpression was seen in 12 out of 68 (18%) of cases (Slomovitz *et al*, 2008). In clear cell endometrial cancer HER2 amplification has been described in two out of nine (22%) and three out of six (50%) of the reported cases (Morrison *et al*, 2006; Grushko *et al*, 2007). Similar controversies exist regarding the clinical relevance of the epidermal growth factor receptor (EGFR) in EC in general and more specifically in type II EC. EGFR expression has been demonstrated in 43–67% of patients with endometrial cancer and its association with clinical outcome has been explored with some studies demonstrating an association between EGFR expression and poor clinical outcome (Khalifa *et al*, 1994; Scambia *et al*, 1994; Niikura *et al*, 1995) whereas others show no association (Reinartz *et al*, 1994). Moreover, the clinical role of EGFR expression specifically in type II EC has not yet been studied. The current study represents a large consecutive series of 134 type II EC cases including 106 patients with uterine serous papillary EC and 28 patients with clear cell EC, who underwent surgery at Mayo Clinic between 1984 and 2004, respectively.

Patients at risk of relapse appear to benefit from adjuvant chemotherapy, and responses to paclitaxel- and platinum-based regimens have been reported in patients with advanced disease (Hoskins *et al*, 2001; Ramondetta *et al*, 2001). Importantly, however, target-based treatment approaches have not yet been explored in type II EC. Recently, we were able to demonstrate significant preclinical activity of a dual HER2 and EGFR kinase inhibitor in endometrial cancer cell lines with HER2 amplification or EGFR expression (Konecny *et al*, 2008). In an effort to obtain a better understanding of the clinical role of HER2 and EGFR in type II EC we studied HER2 gene amplification and EGFR expression in a large cohort of patients with type II EC who were all surgically staged and compared these findings with those seen in a representative cohort of type I endometrioid EC treated during the same time period. These studies were intended to help characterise a subset of type II endometrial cancer patients most likely to benefit from target-based treatment approaches.

## Materials and methods

Upon approval from the Institutional Review Board at Mayo Clinic, we identified 137 patients from our database who underwent surgery for type II endometrial cancer at Mayo Clinic between May 1984 and December 2004. Next, we randomly selected 150 patients who underwent surgery for endometrioid endometrial cancer during the same time period. Of the 287 patients included in this study, 279 patients (97%) had archived paraffin-embedded tissue available for analysis of HER2 gene amplification and EGFR expression. All patients were completely surgically staged and long-term clinical follow up was available for 258 patients (125 patients with type I EC and 133 patients with type II EC). Tissue microarrays were created for each histological subtype. All patients had a hysterectomy and removal of existing adnexal structures and no other malignancy was diagnosed within 5 years before or after the diagnosis of endometrial cancer. Staging was defined according to the International Federation of Obstetricians and Gynecologists (FIGO) surgical staging system. For patients treated before 1988, stage was determined retrospectively on the basis of the surgical and pathologic assessments. The histological classification was according to the World Health Organization classification. Architectural grading was based on the degree of glandular differentiation in accordance with the FIGO guidelines. All surgical procedures were the responsibility of a gynaecologic oncologist. Lymphadenectomy was performed in patients considered by the surgeon to be at risk for lymph node metastasis, according to the histological grade and subtype, as well as primary tumour diameter and the depth of myometrial invasion as determined by an intraoperative analysis of frozen tissue sections. Postoperative adjuvant radiotherapy consisted of external pelvic, para-aortic, or abdominal irradiation or vaginal brachytherapy or a combination of these.

### Tissue specimens and tissue microarray

For tissue microarray construction, a hematoxylin and eosin (H&E) stained histology slide from each patient's archival tumour block was reviewed to identify and mark the location of tumour and normal components. The markings were transferred to the corresponding tissue block. Marked donor blocks were cored into the recipient master block according to a grid map with 0.8-mm spacing from the center of one core to the center of the next core either manually for the type I endometrial cancer tissue microarrays (TMAs) or by use of an automated Beecher ATA 27 Tissue Arrayer for the type II endometrial cancer TMAs (Beecher Instruments Inc., Silver Spring, MD, USA). The tissue microarray blocks constructed from the study tumours incorporated either one tumour core and one normal tissue core (type I endometrial cancer TMAs) or up to three tumour cores (type II endometrial cancer TMAs) from an archival block for each subject. Each core was 0.6 mm in diameter. Cores were arrayed into grids of either 100 cores (type I endometrial cancer TMAs) or 300 cores (type II endometrial cancer TMAs) in master blocks. After construction, tissue microarray blocks were sealed with paraffin and stored at 4°C. Sections (5 *μ*m thick) were cut from the tissue microarray master blocks, mounted on superfrost slides, and assayed for HER2 gene amplification and EGFR expression. HER2 FISH assays were performed using the PathVysion assay (Abbott-Vysis Inc., Des Plaines, IL, USA) as described elsewhere (Press *et al*, 2002). A slide was stained with H&E to confirm the presence of invasive tumour. The corresponding area of invasive carcinoma was enumerated on the FISH slides after hybridisation was complete. Slides were evaluated for HER2 gene amplification by determining the HER2/CEP17-signal ratio in at least 20 tumour nuclei as required by the FDA original approval. If the ratio was <2.0, the specimen was considered to lack gene amplification. For ratios near the cutoff value (i.e., 1.8–2.2), an additional 20 nuclei were evaluated by the same analyst and the ratio was recalculated. In these cases a second analyst also scored at least 40 tumour cell nuclei and if the ratios were both in agreement, the case recorded. All assessments of HER2 status were made by a board-certified pathologist with extensive experience in HER2 FISH testing (MFP). EGFR expression was assessed by IHC and EGFR staining was solely assessed in the glandular components of the endometrial tumour tissue by a board-certified pathologist specialised in gynaecologic malignancies (GLK). A polyclonal rabbit antibody to the C-terminus epitope of EGFR was obtained from Santa Cruz Biotechnology, Santa Cruz, CA, USA (cat. no. 1005; dilution 1 : 250). Tissue sections were microwave-heated for 5 min in an 800-W oven in citrate buffer (0.1 mM, pH 6.0). Sections were then incubated at room temperature overnight with primary antibody. Immunostaining was performed with the avidin–biotin complex method (Vector, Burlingame, CA, USA). Negative controls consisted of substituting normal serum for primary antibodies. For tissue microarray analysis images of H&E and EGFR stains were scanned with the Bliss Imaging System (Bacus Laboratories, Inc, Lombard, IL, USA). The *x* and *y* coordinates of each core within the grid were determined by the software and included as part of the unique identifier, which was linked to the clinical database. Only staining of the tumour cell membranes was considered positive. Immunoreactivity was qualitatively scored by interpreting the staining intensity (negative; weak, moderate, or strong staining) and the percentage of positive tumour cells per core (⩽25%; >25–50%; >50–75%; and >75%). Tissues were graded positive for EGFR expression with ⩾ moderate staining intensity in >25% of the cells examined.

### Statistics

Overall survival was defined as the time to death from any cause. Patients were censored on the date of last contact if a treatment failure event had not been observed. Unadjusted survival was assessed by the Kaplan–Meier method. Log-rank statistic was used for outcome comparison in univariate analysis. Cox regression analysis was used to estimate hazard ratios and their 95% confidence intervals in multivariate analysis adjusted for histology type, FIGO stage, tumour grade, and age. All reported *P*-values and confidence intervals are from two-sided tests. Because well-established and replicated cutoffs for the expression status of EGFR in endometrial cancer were not available, we made the *a priori* choice to analyse and report the scores as dichotomised values.

## Results

**Figure 1:**
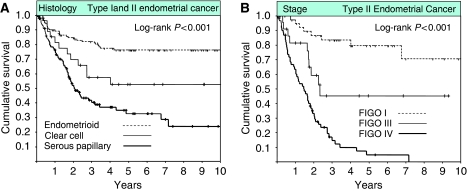
(**A**) Kaplan–Meier survival plots of all type I and type II endometrial cancer patients with available clinical follow-up information (*n*=258) according to the histology type and (**B**) Kaplan–Meier survival plots among type II endometrial cancer patients with available follow up (*n*=133) according to FIGO stage.

The current study is an observational study in which we compared the pattern of HER2 gene amplification and EGFR expression between a cohort of 134 consecutive patients with type II ECs (106 uterine serous papillary and 28 clear cell type histologies) and a representative group of 145 patients with type I EC treated over the same time period. Patients with clear cell and uterine serous papillary histology had a significantly worse OS when compared with endometrioid EC cases (Figure 1A) and in patients with type II EC FIGO stage was a significant predictor of OS (Figure 1B). Tissue microarrays were constructed from the 279 available tumour specimens for each histological subtype and HER2 as well as EGFR status were assessed by FISH and IHC, respectively. The use of tissue microarrays allowed us to obtain technically evaluable results for HER2 in 275 (99%) and for EGFR in 255 (91%) of the 279 patients, respectively (Table 1). HER2 gene amplification was significantly higher in type II EC when compared with type I EC (17 *vs* 1.4%, *P*<0.001). HER2 gene amplification was seen in 18 (17%) of the 105 evaluable uterine serous papillary EC specimens, and in four (16%) of the 25 evaluable clear cell EC specimens. Type I EC demonstrated a lower than expected rate of HER2 gene amplification (1.4%). Furthermore, we were not able to detect an increase in the rate of HER2 gene amplification from grade 1 to grade 3 endometrioid ECs (Table 2). The mean HER2/CEP17 signal ratio was 3.61 (range, 2.00–8.89) in samples with HER2 gene amplification. The mean number of HER2 signals per cell, mean number of CEP17 signals per cell, and the HER2/CEP17 ratios for each HER2-positive case are shown in Table 3.

**Figure 2:**
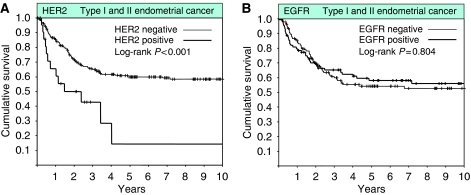
Kaplan–Meier survival plots of all type I and type II endometrial cancer patients with available clinical follow-up information (*n*=258) according to HER2 status (**A**), and EGFR status (**B**).

**Figure 3:**
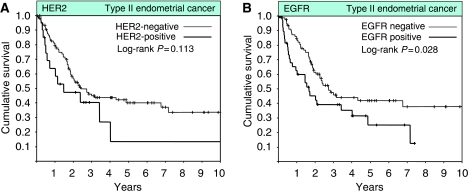
Kaplan–Meier survival plots of all type II endometrial cancer patients with available clinical follow-up information (*n*=133) according to HER2 status (**A**), and EGFR status (**B**).

In contrast to the higher rate of HER2 gene amplification in type II EC, EGFR expression was significantly lower in type II EC compared with type I EC (34 *vs* 46%, *P*=0.041; Table 1). Moreover, the rate of EGFR expression was significantly lower in grade 3 compared with grade 1/2 endometrioid EC (31 *vs* 52%, *P*=0.016). When analysing all 279 patients, there was a significant increase in the rate of HER2 amplification, but not of EGFR expression, from stage I to stage IV disease (HER2, *P*=0.025; EGFR, *P*=0.667; Table 2). Similarly, we found a significant increase in the rate of HER2 gene amplification from grade 1 to 3 tumours, and conversely a significant decrease in the rate of EGFR expression from grade 1 to 3 tumours (*P*=0.028 and *P*=0.016, respectively; Table 2). HER2 gene amplification was associated with significantly worse OS in univariate analysis (HER2, *P*<0.001; Figure 2A) but did not retain independent prognostic significance when accounting for grade, stage, age, and histology (HER2, *P*=0.860). EGFR was not prognostically relevant in the entire cohort of 279 patients including both type I and Type II EC (*P*=0.804; Figure 2B). When analysing only patients with type II endometrial cancer EGFR expression, but not HER2 gene amplification, was statistically significantly associated with worse OS when compared with those patients with either non-HER2 amplified or non-EGFR expressing EC (HER2, median OS 18 *vs* 29 months, *P*=0.113; EGFR, median OS 20 *vs* 33 months, *P*=0.028; Figure 3A and B). EGFR-retained independent prognostic significance for OS in type II EC when accounting for age and stage in multivariate analysis (EGFR, risk ratio 1.81, 95% CI 1.10 – 2.99, *P*=0.0197; Table 4).

## Discussion

Our understanding of the pathogenesis or the optimal treatment of uterine serous papillary and clear cell EC is limited. The existing lack of prospective clinical trials assessing adjuvant therapy in these aggressive variants of EC and the absence of targeted treatment approaches reflects at least in part the low incidence of type II EC with the accompanying limited single institutional experiences. However, a reasonable estimate would predict that 3000–4000 women in the United States alone will be diagnosed with type II EC during 2008 and an estimated 55–65% will die, accounting for approximately 18–24% of all endometrial cancer-related deaths (Podratz and Mariani, 2003). Thus the absence of randomised clinical trials for one of the most aggressive gynaecologic malignancies appears unacceptable.

Evidence for a role of HER2 and EGFR in the pathogenesis of various cancers has led to the rational design and development of agents that selectively target HER2 and EGFR. In unselected patients with endometrial cancer, HER2 amplification/overexpression represents a rare event. However, the findings of our study confirm and extend previous reports that indicate HER2 amplification/overexpression is seen more commonly in well-defined subtypes of EC such as uterine serous papillary cancer or clear cell cancer. Trastuzumab (Herceptin) a humanised anti-HER2 antibody has recently been approved for the adjuvant treatment of HER2-overexpressing (3+ IHC) or FISH-positive primary breast cancers based on a highly significant 52% reduction in the risk of recurrence in node-positive HER2-positive primary breast cancer (Romond *et al*, 2005). More recent advances in biotechnology have led to the development of the oral dual tyrosine kinase inhibitor lapatinib, which has been shown to have significant activity in trastuzumab-refractory breast cancer (Geyer *et al*, 2006). Encouraged by these clinical response data which were generated in breast cancer patients with HER2 amplification/overexpression, we comprehensively assessed the rate of HER2 gene amplification in a large consecutive series of patients with uterine serous papillary and clear cell endometrial cancers. These studies were intended to define a subset of type II EC patients who may benefit from a selective HER2 inhibitor or a dual kinase inhibitor, which targets both HER2 and EGFR. The aggressive nature of type II EC is confirmed in our study by the observation that two out of three patients diagnosed with uterine serous papillary and one of two diagnosed with clear cell cancer had extra-uterine disease at the time of primary surgery. HER2 gene amplification was found in 17 and 16% of the serous papillary and clear cell EC cases, respectively. Earlier studies have reported higher rates of HER2 gene amplification in uterine serous papillary EC. The most recent study conducted by Grushko *et al*. detected HER2 gene amplification in 6 out of 28 (21%) patients with serous papillary and 3 out of 6 (50%) of clear cell EC (Grushko *et al*, 2008). This study cohort of that report, however, differed considerably from the consecutive series in our study, as it included patients with measurable stage III, stage IV, or recurrent endometrial cancer that were enrolled in GOG study no. 177 evaluating the role of doxorubicin and cisplatin with or without paclitaxel in advanced endometrial cancer. Santin *et al*. reported HER2 gene amplification in 14 out of 30 (47%) patients with uterine serous papillary cancer. Importantly, however, of the 30 patients included in his study, 12 were African-American patients of whom eight (67%) showed amplification by FISH compared with six (33%) of the remaining 18 Caucasian patients. Information on the patient's race was not collected in our current study, yet possible differences in patient populations may account for the reported discrepancy in the rate of HER2 gene amplification between both studies. Importantly, previous studies have reported a higher incidence of serous papillary endometrial cancer and a higher rate of HER2 gene amplification in African-American patients when compared with Caucasian patients (Maxwell and Risinger, 2006; Morrison *et al*, 2006). When combining the results of the aforementioned studies, which have all used FISH for assessment of HER2 status, HER2 gene amplification, at average, was detected in 54 out of 222 (24%) patients with serous papillary EC. In contrast to our low rate of HER2 gene amplification in type I EC, other groups have been able to demonstrate HER2 gene amplification in 9 out of 363 (4%) of unselected type I EC and in 5 out of 63 (8%) grade 3 endometrioid endometrial cancers (Morrison *et al*, 2006). We may not have been able to confirm these rates possibly because of differences in the patient populations or because of the smaller sample size of type I ECs in our study. Previous data on the rate of HER2 gene amplification in clear cell EC (22–50%) are unquestionably limited by the small number of samples investigated so far. The actual rate of HER2 gene amplification in clear cell EC may thus be somewhat lower according to the findings of our study.

The clinical role of EGFR has not been studied well in EC, moreover this is the first study to evaluate the incidence and prognostic relevance of EGFR expression in type II EC. Importantly, EGFR may have a dual role in EC, such that high EGFR expression in type I EC was associated with low grade and favourable outcome. In contrast, EGFR expression in type II EC was associated with high grade and adverse clinical outcome. Therefore, EGFR expression did not appear to impact disease progression in well-differentiated endometrioid endometrial cancer, but did seem to affect disease progression in undifferentiated nonendometrioid endometrial cancer. To date EGFR inhibitors have not been clinically tested in type II EC. Importantly, the clinical benefit observed with anti-EGFR tyrosine kinase inhibitors (TKIs) across different disease entities has been variable. For example, EGFR TKIs are largely inactive in colorectal cancer and breast cancer (Tan *et al*, 2004; Baselga and Arteaga, 2005). Nevertheless, two of these drugs, gefitinib and erlotinib, have demonstrated clinical activity in non small cell lung cancer and responses have been observed in patients with advanced pancreatic cancer and in head-and-neck cancer (Baselga, 2006). Preclinical data suggest that EGFR inhibitors may be clinically active in well-defined subgroups of endometrial cancer patients with HER2 gene amplification or high levels of EGFR expression (Konecny *et al*, 2008). Importantly, however, EGFR receptor expression levels when assessed by IHC have not been able to predict a response to EGFR inhibitors in other tumour types. Earlier clinical studies in other disease entities show that potential markers of sensitivity to EGFR TKIs include the presence of EGFR gene amplification, mutations of the EGFR gene, and increased expression of EGFR ligands (Baselga and Arteaga, 2005). Earlier studies have demonstrated significantly higher expression levels of the EGFR ligands TGF-*α* and amphiregulin in EC compared with normal endometrium (Pfeiffer *et al*, 1997; Ejskjaer *et al*, 2007). The roles of EGFR gene amplification or mutations in EC, however, have not yet been studied.

Although HER2 gene amplification or EGFR expression each can only be detected in small subsets of patients with type II EC, collectively, 46% of the patients with type II EC demonstrated HER2 gene amplification and/or EGFR expression in our study. The pooling of national or global patient resources should allow the realisation of prospective clinical trials (that stratify for HER2 gene amplification or EGFR expression) for patients with type II EC that may involve HER2 and EGFR tyrosine kinase inhibitors in well-defined subsets with HER2 gene amplification or EGFR expression.

## Figures and Tables

**Table 1:** Patient and disease characteristics of endometrial cancer patients with endometrioid, uterine serous papillary, and clear cell type histology

	**Endometrioid**		**Uterine serous papillary**		**Clear cell**	
	**No.**	**Valid %**	**No.**	**Valid %**	**No.**	**Valid %**
	145		106		28	
Median age (range)	65 (38–90)		68 (47–93)		68 (41–86)	
Median follow up (months)	83 (0.3–270)		20 (0.1–162)		38 (0.2–180)	
Surgery dates (range)	3/84–3/04		2/84–12/04		3/88–5/04	
						
*Stage*						
I	97	(67)	30	(29)	13	(46)
II	11	(8)	0	(0)	0	(0)
III	22	(15)	17	(17)	7	(25)
IV	14	(10)	56	(54)	8	(29)
						
*Grade*						
1	51	(35)	1	(1)	2	(7)
2	54	(37)	1	(1)	4	(14)
3	40	(28)	87	(98)	22	(79)
						
*HER2 Status*						
Positive	2/143	(1.4)	18/105	(17)	4/25	(16)
						
*EGFR status*						
Positive	60/130	(46)	36/101	(36)	6/24	(25)

**Table 2:** Associations between HER2 gene amplification or EGFR expression and disease characteristics including type of histology, FIGO stage, and grade

	**Patients**		**HER2-positive**		**EGFR-positive**	
	**No.**	**Valid %**	**No.**	**Valid %**	**No.**	**Valid %**
Histology*						
Endometrioid grade 1	51	(18)	1/50	(2)	27/48	(56)
Endometrioid grade 2	54	(20)	1/53	(2)	22/47	(47)
Endometrioid grade 3	40	(15)	0/40	(0)	11/35	(31)
Clear cell	28	(10)	4/25	(16)	6/24	(25)
Serous papillary	106	(37)	18/105	(17)	36/101	(36)
						
FIGO stage**						
I	140	(52)	8/140	(6)	54/127	(43)
II	11	(4)	0/11	(0)	4/9	(44)
III	46	(17)	3/46	(7)	14/43	(33)
IV	77	(27)	13/77	(17)	27/72	(38)
						
Grade***						
1	54	(21)	1/54	(2)	28/51	(55)
2	59	(23)	2/59	(3)	23/52	(44)
3	148	(56)	17/148	(11)	45/138	(33)

Unknown data: grade (*n*=17), stage (*n*=4), HER2 (*n*=6), EGFR (*n*=24).

^*^ *χ*^2^ test type I versus type II: *P*<0.001 for HER2, and *P*=0.041 for EGFR.

^**^ *χ*^2^ test: *P*=0.025 for HER2, and *P*=0.667 for EGFR.

^***^ *χ*^2^ test: *P*=0.028 for HER2, and *P*=0.016 for EGFR.

**Table 3:** Mean number of HER2 and Cep17 signals per cell, as well as the HER2/Cep17 ratios for each HER2-positive case

**HER2/Cep17 ratio**	**HER2 signals/cell**	**Cep17 signals/cell**	**Histology**
2.00	3.40	1.70	USPC
2.03	3.95	1.95	CC
2.05	5.73	2.80	USPC
2.06	3.40	1.65	USPC
2.06	5.57	2.70	CC
2.08	5.00	2.40	USPC
2.28	4.45	1.95	USPC
2.55	6.75	2.65	USPC
2.63	5.4	1.55	USPC
2.71	3.25	1.20	USPC
2.88	4.75	1.65	USPC
2.98	5.95	2.00	USPC
3.16	8.05	2.55	USPC
3.48	5.40	1.55	CC
3.93	5.70	1.45	USPC
3.98	8.95	2.25	USPC
4.21	13.25	3.15	USPC
4.23	8.25	1.95	E
4.41	7.50	1.70	USPC
4.58	13.75	3.00	USPC
6.59	10.55	1.60	E
7.15	17.55	2.45	CC
8.89	15.55	1.75	USPC

USPC=uterine serous papillary cancer; CC=clear cell cancer; E=endometrioid endometrial cancer.

Missing data: *n*=1.

**Table 4:** Prognostic significance of EGFR status in type II endometrial cancer patients using multivariate analysis

**Parameter**	**Risk ratio (95% CI)**	**Wald test**
Age	1.06 (1.03, 1.09)	*P*=0.0002
		
*Stage*		
1–2 (reference)	1.0	
3	3.23 (1.24, 8.40)	*P*=0.0160
4	11.8 (5.33, 26.2)	*P*<0.0001
		
*EGFR*		
Negative (reference)	1.0	
Positive	1.81 (1.10, 2.99)	*P*=0.0197

A stepwise method was used for variable selection. EGFR expression, stage, and age were selected as significant prognostic factors.
